# The Association between Physical Fitness Performance and Subjective Happiness among Taiwanese Adults

**DOI:** 10.3390/ijerph17113774

**Published:** 2020-05-26

**Authors:** Hui-Ling Chen, Po-Fu Lee, Yun-Chi Chang, Fu-Shu Hsu, Ching-Yu Tseng, Xin-Yu Hsieh, Chien-Chang Ho

**Affiliations:** 1 Graduate Institute of Educational Leadership and Development, Fu Jen Catholic University, New Taipei City 242, Taiwan; 024145@mail.fju.edu.tw; 2 Graduate Institute of Sport Coaching Science, Chinese Culture University, Taipei City 111, Taiwan; 3 Department of Physical Education, Fu Jen Catholic University, New Taipei City 242, Taiwan; 142498@mail.fju.edu.tw (Y.-C.C.); 018272@mail.fju.edu.tw (F.-S.H.); 015844@mail.fju.edu.tw (C.-Y.T.); fj03684@mail.fju.edu.tw (X.-Y.H.); 4 Department of Physical Therapy and Assistive Technology, National Yang-Ming University, Taipei City 112, Taiwan; 5 Research and Development Center for Physical Education, Health, and Information Technology, Fu Jen Catholic University, New Taipei City 242, Taiwan

**Keywords:** physical activity, health related physical fitness, quality of life, mental health

## Abstract

The purpose of the present study was to determine the association between health-related physical fitness (HRPF) performance and perceived happiness status among adults in Taiwan. A cross-sectional study was conducted, and data derived from the National Physical Fitness Survey in Taiwan 2014–2015 were reviewed. The participants included 27,930 men and 30,885 women, aged 23 to 64 years. Each participant completed a standardized, structured questionnaire and underwent anthropometric variable and HRPF measurements. The happiness outcome of an individual was obtained using the questionnaire, and the results were stratified into happy (very happy, quite happy, and fair) and unhappy (unhappy and not at all happy) groups for perceived happiness status. HRPF measurements were evaluated using cardiorespiratory endurance (3 min step test), muscle strength and endurance (1 min sit-up test), flexibility (sit-and-reach test), and body composition (body mass index (BMI) and waist-to-hip ratio). To determine the existence of a dose–response relationship between HRPF component levels and happiness status, four quartiles of HRPF components were analyzed using multiple logistic regression models. Multiple logistic regression results indicated that with the worst performance level of HRPF components as a baseline, significant associations were observed for the sit-and-reach test (third level: OR = 1.24, 95% CI: 1.02–1.49) and BMI (second level: OR = 0.78, 95% CI: 0.64–0.95) among men. For women, significant associations were observed for the 1 min sit-up test (second level: OR = 1.28, 95% CI: 1.03–1.60; third level: OR = 1.32, 95% CI: 1.04–1.67; fourth (the best) level: OR = 1.48, 95% CI: 1.12–1.95) and BMI (third level: OR = 0.73, 95% CI: 0.58–0.92). The current study suggested that higher values in flexibility and body composition, happiness-related factors, potentially improve the occurrence of happiness among men. Moreover, this positive effect of higher values of muscle strength, endurance, and BMI was observed for the occurrence of happiness in women. However, the relevant mechanism underlying this phenomenon must be further explored.

## 1. Introduction

Discussions of the worst economic crisis of the past decades have given rise to discussions of happiness; the topic of happiness is frequently being addressed because it indicates the state of the economic progress of the nation as well as the physical and mental health of the citizens. According to the Global Burden of Disease Study in 2010, mental and behavioral disorders accounted for 22.9% of the years lived with disability global burden, which is the highest among all categories, thus warranting further study and solutions [1].

Responding to the aforementioned effects, studies have first focused on the preventive and curative approaches toward negative mental health disorders [2]. Physical activity participation positively affects depression and anxiety [3,4,5]. However, the evidence of these reciprocal relationships between physical activity and mental health constructs are rare. From the perspective of positive psychology, few published studies have focused on disease prevention rather than the promotion of positive mental health outcomes, such as happiness. The positive thought and opportunity of health promotion models may be identified by examining physical activity participation and positive components of mental wellbeing [6], and may provide answers to some crucial questions.

Participating in physical activity, such as exercise and sports, can cause joy [7] and promote health, which are crucial for a happy life. Such occurrences are conventionally partially attributed to the release of endorphins, effects of social interaction, and perceived self-esteem through successful performance [8]. Furthermore, based on a large body of literature, Fox [9] summarized the potential of physical activity as a means of upgrading life quality and reported a possible mechanism of how it enhances mental wellbeing (e.g., enhances self-esteem, improves mood states, reduces state and trait anxiety, improves resilience to stress, and improves sleep). Thus, because physical activity improves mental wellbeing and mitigates the negative effects of depression and anxiety, physical activity can affect happiness accordingly [7].

In such scenarios, studies have generally analyzed how physical activities can affect happiness. For example, Hyde et al. [10] reported that emerging adults (19.3 ± 2.8 years) who were more physically active were inclined to report more pleasure-activated feelings (e.g., happiness) than those who were less active. The same result was provided by Piqueras et al. [11], which indicated that happiness is positively associated with health behaviors through biological responses to stress and with maintaining healthy lifestyles when participating in physical activities. Wang et al. [12] examined the long-term effect of physical activity participation on happiness. The results showed that physical activity (leisure-time) was associated with reduced odds of unhappiness after 2 to 4 years and exhibited a positive long-term effect on happiness. However, although the relationship between physical activity and happiness has been established by researchers, nearly all studies have focused on the frequency of participation in physical activity instead of their participation level (achievement) or fitness states, particularly health-related physical fitness (HRPF).

Few studies have considered the types of physical activities that enable participants to improve their happiness levels. Mohammadi et al. [13] indicated the gap in the field and investigated the relationship between perceived happiness and different fitness levels (perceived). The results showed significant differences, in that physically active groups exhibited a higher level of happiness than semi-active and passive groups. Nevertheless, the study, on the basis of a particular group (university students) with data from the pencil-and-paper tests, appeared to be limited by its design for explaining the situation in a general population (adults). A study with an overall age of adults employing a straightforward method is required to evaluate the fitness status of adults.

The National Physical Fitness Survey of Taiwan on HRPF provides opportunities to revalidate the existing knowledge and to explore the unknown phenomenon. The survey not only included a structural test on HRPF but also collected behavioral and psychological information of participants, such as perceived happiness status. Based on this existing advantage, the current study aimed to determine the association between HRPF and perceived happiness status among Taiwanese adults by analyzing the database from a nationwide survey.

## 2. Materials and Methods

### 2.1. Study Design and Participants

In this cross-sectional study, the data sources were obtained from the National Physical Fitness Survey (2014–2015), which was a nationwide survey in Taiwan that was conducted to examine the HRPF performance of Taiwanese individuals between October 2014 and March 2015. This survey was conducted by the Sports Administration, Ministry of Education in Taiwan. The details of the design, sampling protocols, and data validation of the series of annual surveys have been previously described [14]. All participants were recruited through stratified convenience sampling from 46 physical fitness test stations in 22 cities and counties in Taiwan. The study protocol comprised three phases: filling in a standardized structured questionnaire, pretest health screening, and HRPF tests. Each participant was interviewed based on this questionnaire, which included items related to demographic characteristics, socioeconomic status, lifestyle behaviors, current health status, and associated factors related to happiness. The HRPF tests were then conducted after pretest health screenings (screening for health limitations affecting the eligibility for participation in HRPF tests), which included measures of the resting heart rate (HR) and blood pressure as well as completing a modified Physical Activity Readiness Questionnaire [15]. Finally, a total of 58,815 Taiwanese adults aged between 23 and 64 years were included in this analysis. These data comprised deidentified secondary data, which were released to the public for research purposes [14]. This study was approved by the Institutional Review Board of Chung Shan Medical University Hospital (CRREC-104-015), registered on 18 October 2016. Each participant provided an informed consent form after completely understanding the survey.

### 2.2. Data Collection

A standardized, structured questionnaire was used to collect data on demographic characteristics (i.e., age and gender), socioeconomic status, (i.e., education, monthly income, and marital status), lifestyle behaviors (i.e., smoking, betel-nut chewing, and dieting), and the perceived health status of subjects through face-to-face interviews. Anthropometric variables included body weight, height, waist circumference (WC), and hip circumference (HC) measurements; body mass index (BMI, in kilograms per square meter); and waist-to-hip ratio (WHR). The cut-off BMI points for obesity status were adopted as suggested by the Health Promotion Administration, Ministry of Health and Welfare in Taiwan, namely underweight (BMI < 18.5 kg/m^2^), normal weight (18.5 ≤ BMI < 24 kg/m^2^), overweight (24 ≤ BMI < 27 kg/m^2^), and obese (BMI ≥ 27 kg/m^2^) categories [16]. Blood pressure (systolic (SBP) and diastolic (DBP)) and HR were measured for safe preliminary screening after a resting period of at least 5 min.

### 2.3. Measure of Happiness

Happiness was measured using a questionnaire that has been described previously [17]: “Taking all things together, would you say you are, on the whole: Very happy, quite happy, fair, unhappy, or not at all happy?”. Because of the low percentage of responses in the “not at all happy” category (0.3%), the responses were combined into a binomial outcome, happy (i.e., very happy, quite happy, and fair) and unhappy (i.e., unhappy and not at all happy) in this analysis.

### 2.4. Measures of Health-Related Physical Fitness

HRPF was evaluated based on cardiorespiratory endurance, muscle strength and endurance, flexibility, and body composition as measurements through a 3 min step test, 1 min sit-up test (reps/min), sit-and-reach test (cm), BMI (kg/m^2^), and WHR of Taiwanese adults aged 23–64 years [18]. These HRPF measurements were conducted by trained examiners, who attended a regional training seminar and passed a certification test on standardized procedures, which have been reported in previous studies [14,19].

Cardiorespiratory endurance: Cardiorespiratory endurance was measured using the 3 min-step test in the present study and was adapted from the Harvard Step Test [20], which assessed the cardiorespiratory fitness of individuals based on the speed of HR recovery from the submaximal exercise test. Participants performed 24 steps/min for 3 min based on the protocol for the step test, maintaining the same stepping rate on a 35 cm high step [21]. The subjects were assisted by the examiner and a metronome cadence for proper stepping technique and constant step maintenance. When the step test was completed, the participants were immediately seated, and their heart rates were counted for 1 min, starting within 5 s of the end of the submaximal exercise test. The sum of the heartbeat counts during the recovery period was compared with the sum of the heart rates during three periods after the step test: 1.0–1.5 min, 2.0–2.5 min, and 3.0–3.5 min [18]. The cardiorespiratory endurance index (CEI) was calculated based on the method, which assumes that CEI = duration of step test (s) × 100/sum of heartbeat counts during the recovery period/2.

Muscle strength and endurance: Muscle strength and endurance were measured using the 1 min sit-up test in this study, with the maximum number of sit-ups performed within a period of 1 min [19,22]. Participants lay down on a mat with knees bent at right angles and hands behind the head. The ankles were firmly held by a partner for support and maintaining the counts.

*Flexibility:* Flexibility was measured by the sit-and-reach test in this study, which was scored as the highest distance (in cm) reached on the ruler with the fingertips [19,22,23,24]. The sit-and-reach box had a measuring scale, where 30 cm was at the level of the feet. Before the test, shoes were removed and participants were instructed to slowly reach forward, with their knees completely extended as far as possible on the scale.

Body composition: Body composition was measured using the BMI and WHR in this study. First, the participants removed their shoes and heavy clothes; then, anthropometric evaluations, including of body weight, height, WC, and HC, were performed; then, the BMI and WHR were calculated using the following formulas: BMI = body weight (kg)/height squared (m^2^) and WHR = WC (cm)/HC (cm). The body weight and height were recorded to the nearest 0.1 kg and 0.1 cm with an electronic height–weight scale. The WC was measured to the nearest 0.1 cm with a flexible steel tape measure placed midway between the lowest rib and iliac crest when participants were in a standing position at the end of an exhalation. The HC was measured to the nearest 0.1 cm at the widest part of the hip region in the standing position.

To achieve optimal HRPF performance, participants were asked to avoid any other exercise before the HRPF test. They had 10 min to prepare with a static warmup (i.e., stretches), which was led by the certified physical trainers. All participants performed the measurements in the following order with a sufficient rest period (3–5 min) between the measurements: height, body weight, WC, HC, 1 min sit-up test, sit-and-reach test, and 3 min step test.

### 2.5. Statistical Analyses

Data were analyzed using SAS 9.4 (SAS Institute, Cary, NC, USA). A *t*-test was performed to analyze continuous variables and a chi-square test was used for the analysis of categorical variables. For each HRPF measurement, participants were classified into four quartiles based on their HRPF performance levels to examine the dose–response relationship. The adjusted odds ratios (ORs) with 95% confidence intervals (CIs) for happy status were calculated using unconditional logistic regression models according to quartiles of each HRPF measurement. Values are presented as means ± standard deviation (SD) or frequency percentages. Statistical results were significant with *p* < 0.05.

## 3. Results

A total of 58,815 participants with complete data were included from the nationwide survey in Taiwan (47.49% men). Table 1 shows the demographic characteristics and anthropometric indices. Numerous participants (97%) reported that they were happy with their current lives; that percentage was consistent with the high proportion of happiness responses in the latest worldwide report [25]. All participants were divided into dichotomous groups as unhappy and happy. Significant differences were observed between groups (*p* < 0.05) on all relevant variables, namely age, gender, height, body weight, BMI, WC, HC, WHR, obese status, education, monthly income, marital status, self-reported health status, smoking status, and chewing betel nuts.

Table 2 compares intergroup differences using each HRPF measurement (3 min step test, 1 min sit-up test, sit-and-reach test, BMI, and WHR). In general, without gender differences, all measurements showed significant differences, excluding the 1 min sit-up test. Moreover, differences for the 3 min step test and sit-and-reach test were statistically significant among both men and women. Furthermore, the difference for the 1 min sit-up test was significant among females.

Table 3 represents the multivariate-adjusted ORs of the relationships. Furthermore, the 3 min step test and sit-and-reach test were statistically significant among both men and women before adjustment, and the 1 min sit-up test was significant, particularly among women. This test was the only significant test after confounders were adjusted for. However, the connections between these associations were very low according to the ORs (a range of 1–1.1) before or after adjustments. On the basis of the results, the strong positive effect of physical fitness on happiness was difficult to understand through intergroup comparisons (divided based on the happiness status).

Table 4 presents the results of the logistic regression for further understanding. Participants were divided into four levels based on the quartiles of each measurement. The lowest (commonly worst) level of performance was fixed as a reference in all regression for analysis (OR = 1.00). After factors were adjusted by potential confounders, statistical evidence revealed that male participants, who performed in the second level of the sit-and-reach test (21–29 cm), were more likely to respond that they were happy (0.24 OR against the baseline). An OR of 0.78 was observed in the third level of the male BMI (22.53–24.57 kg/m^2^) (e.g., participants of this group might have a 22% chance (odds) of being unhappy compared with the baseline group). For females, participants with improved performance on the 1 min sit-up test exhibited higher odds of being happy. Females in the optimal performance group exhibited ORs higher than the baseline group, second group, and third group by 0.48, 0.32, and 0.28, respectively. Furthermore, females in the second level of BMI (20.54–22.37 kg/m^2^) presented odds of 0.27 units of being unhappy compared with the baseline (0.73 ORs).

## 4. Discussion

The present study examined the association between HRPF and perceived happiness status. A large amount of survey data was used to identify the HRPF measurements that were more likely to be caused by the effects of physical fitness on happiness. The results revealed that specific performances of HRPF may be potential factors regarding the effects on happiness. For example, the 1 min sit-up test levels of women were positively associated with happiness status. The 1 min sit-up test revealed the strength of abdominal muscles and was partially correlated with the body composition of individuals (abdominal skinfold thickness) and shape, particularly for women [26]. Women of all ages experience suffering and serious cognitive, affective, and behavioral symptoms triggered by body dissatisfaction [27,28]. Having a healthy shape may improve their body image and physical attractiveness, which can develop body satisfaction and subjective happiness [29]. However, a plausible reason for the absence of this finding for men participants is that having low physical fitness (particularly abdominal muscle strength) is socially acceptable (because of age, marriage, and wining and dining clients).

Compared with the baseline, overweight male participants (BMI 24.28–26.81 kg/m^2^) and pre-overweight female participants (BMI 20.54–22.37 kg/m^2^) are less likely to be happy. This result indicated that being overweight (men) or the possibility of becoming overweight (women) may be associated with anxiety, depression [30,31], low self-esteem [32], and other negative psychological influences among the Taiwanese adult population. However, the baseline groups (BMI > 26.81 kg/m^2^ for men and > 24.80 kg/m^2^ for women) were more likely to be happy. This finding is interesting in light of existing knowledge.

Researchers have indicated the negative effects of overweight and obesity on subjective wellbeing [33,34]. However, according to the literature in psychology and economics, subjective wellbeing has been used as an overarching term to encompass indicators of self-reported happiness, general satisfaction with life, and general quality of life [35]. Bockerman et al. [36] demonstrated an interesting finding to explain this inconsistency. According to their analysis, limited evidence is available for the adverse effects of obesity on subjective wellbeing when health and functional status are controlled for. People who maintain a suitable health condition and who are without functional disability may experience lower-than-expected adverse effects from obesity (or even overweight). Accordingly, the participants in the present study were preliminarily examined before data collection (blood pleasure and safety questionnaire), thus largely eliminating the aforementioned population and mediating the effect of the health status and disability as a consequence.

Although little direct evidence is available on the relationship between physical flexibility and happiness, one result reported that flexibility (the sit-and-reach test) positively affected happiness from the second highest performance group (21–29 cm) of men (a positive OR of 0.24 was shown). From an injury perspective, flexibility is related to health status, particularly back pain, muscle soreness, and muscle injury [37]. Decreasing body pain can decrease adverse experiences and lead to a happier life. However, further examination is needed.

The present study has several advantages. First, the data were representative of the Taiwanese population, which eliminated selection biases from the sampling. Second, according to the review of the relevant literature, this is the first study to investigate the relationship between HRPF performances and subjective happiness. However, this study has some limitations. First, the dichotomous system of subjective happiness might miss valuable information from the personal experiences of participants. Additionally, the prominent happiness level of Taiwanese citizens [25] causes a hugely imbalanced dataset for this study. The difference between happy and unhappy groups is strongly divergent and may result in often described significances. Future investigations should examine the relationship between physical fitness levels and happiness in, or compared with, populations from different cultures, societies, or even religions in order to provide a comprehensive view. Second, a causal relationship cannot be demonstrated because of the cross-sectional study design. Third, as mention previously, the participants were preliminarily examined for their health status. The survey data eliminated the functionally disabled and physically unfit population. Besides, due to the limited information that can be used, we suggest the authorities in Taiwan should consider a more informative questionnaire, such as the International Physical Activity Questionnaire (IPAQ) or the Global Physical Activity Questionnaire (GPAQ) in future investigations, in order to upgrade the value of this database.

## 5. Conclusions

In conclusion, our study has performed a preliminary investigation of the relationship between HRPF performance and subjective happiness among Taiwanese healthy adults. This study showed noteworthy results regarding positive effects (especially with the 1 min sit-up test, BMI, and sit-and-reach test) on subjective happiness. Future research may investigate the causal relationships and their possible mechanisms.

## Figures and Tables

**Table 1:** Demographic characteristics and anthropometric indices of the study population.

Variables	Perceived Happiness Status		*p*-Value
	Unhappy (*n* = 1874)	Happy (*n* = 56,941)	
Age (y)	39.50 ± 11.23	41.55 ± 11.76	<0.001 *
Gender (% men)	54.64%	47.25%	<0.001 *
Height (cm)	165.26 ± 8.51	164.18 ± 8.64	<0.001 *
Body Weight (kg)	65.81 ± 12.87	64.39 ± 12.13	<0.001 *
BMI (kg/m^2^)	23.99 ± 3.70	23.78 ± 3.45	0.018 *
Obese Status (%)			0.006 *
Underweight	3.47%	4.09%	
Normal Weight	50.75%	51.61%	
Overweight	25.56%	27.01%	
Obese	20.22%	17.29%	
WC (cm)	80.88 ± 10.43	79.92 ± 9.79	<0.001 *
HC (cm)	95.95 ± 6.88	95.46 ± 6.58	0.003 *
WHR	0.84 ± 0.07	0.84 ± 0.07	<0.001 *
Education (%)			0.006 *
Elementary School or Lower	3.28%	3.62%	
Junior or Senior School	24.67%	27.86%	
College or Higher	72.05%	68.52%	
Monthly Income (%)			<0.001 *
≦20,000 NTD	24.86%	20.90%	
20,001–40,000 NTD	40.79%	41.16%	
≧40,001 NTD	34.35%	37.94%	
Marital Status (%)			<0.001 *
Never Married	50.46%	54.24%	
Married	43.44%	42.08%	
Divorced/Widowed/Other	6.10%	3.69%	
Self-Reported Health Status (%)			<0.001 *
Excellent or Good	18.97%	62.16%	
Fair	37.04%	32.48%	
Very Bad or Poor	43.99%	5.36%	
Smoking Status (%)			<0.001 *
Never	76.83%	83.98%	
Current	17.23%	10.68%	
Former	5.94%	5.33%	
Chewing Betel Nuts (%)			<0.001 *
Never	91.55%	95.11%	
Current	3.75%	1.99%	
Former	4.71%	2.90%	

Abbreviations: BMI, body mass index; HC, hip circumference; NTD, new Taiwan dollar; SD, standard deviation; WC, waist circumference; WHR, waist-to-hip ratio. Values are expressed as means ± SD. * Significantly different between happy and unhappy groups according to Student’s *t*-test or chi-square test at *p* < 0.05.

**Table 2:** The comparison of health-related physical fitness measurements between happy and unhappy groups in Taiwanese adults.

Variables	Perceived Happiness Status		*p*-Value
	Unhappy (*n* = 1874)	Happy (*n* = 56,941)	
**Men (*n* = 27,930)**			
3 min step test	56.22 ± 10.41	57.70 ± 10.48	<0.001 *
1 min sit-up test (reps/min)	28.15 ± 10.88	28.74 ± 10.19	0.088
Sit-and-reach test (cm)	20.28 ± 10.96	21.63 ± 10.62	<0.001 *
BMI (kg/m^2^)	24.91 ± 3.63	24.76 ± 3.24	0.176
WHR	0.87 ± 0.06	0.87 ± 0.06	0.464
**Women (*n* = 30,885)**			
3 min step test	54.88 ± 12.72	56.00 ± 11.57	0.011 *
1 min sit-up test (reps/min)	16.73 ± 10.39	17.92 ± 10.52	0.001 *
Sit-and-reach test (cm)	25.85 ± 11.07	27.50 ± 11.05	<0.001 *
BMI (kg/m^2^)	22.87 ± 3.46	22.91 ± 3.39	0.758
WHR	0.80 ± 0.07	0.80 ± 0.07	0.829
**Total (*N* = 58,815)**			
3 min step test	55.62 ± 11.53	56.81 ± 11.10	<0.001 *
1 min sit-up test (reps/min)	22.97 ± 12.08	23.03 ± 11.69	0.829
Sit-and-reach test (cm)	22.81 ± 11.35	24.73 ± 11.24	<0.001 *
BMI (kg/m^2^)	23.99 ± 3.70	23.78 ± 3.45	0.018 *
WHR	0.84 ± 0.07	0.84 ± 0.07	0.001 *

Abbreviations: BMI, body mass index; SD, standard deviation; WHR, waist-to-hip ratio. Values are expressed as means ± SD. * Significantly different between happy and unhappy groups according to Student’s *t*-test at *p* < 0.05.

**Table 3:** Multivariate-adjusted ORs for happiness’ relation to health-related physical fitness measurements after adjustment for potential confounders.

Variables	Model 1 (Unadjusted)			Model 2 (Adjusted ^a^)		
	OR	95% CI	*p*	OR	95% CI	*p*
**Men (*n* = 27,930)**						
3 min step test	1.01	1.01–1.02	<0.001 *	1.00	1.00–1.01	0.662
1 min sit-up test (reps/min)	1.00	1.00–1.01	0.398	1.00	0.99–1.01	0.833
Sit-and-reach test (cm)	1.01	1.01–1.02	0.001 *	1.00	1.00–1.01	0.202
BMI (kg/m^2^)	0.99	0.97–1.01	0.276	1.01	0.99–1.04	0.267
WHR	1.88	0.51–6.89	0.339	1.37	0.32–5.82	0.668
**Women (*n* = 30,885)**						
3 min step test	1.01	1.00–1.01	0.011 *	1.00	0.99–1.00	0.332
1 min sit-up test (reps/min)	1.01	1.00–1.02	0.008 *	1.01	1.00–1.02	0.015 *
Sit-and-reach test (cm)	1.01	1.01–1.02	0.001 *	1.01	1.00–1.01	0.110
BMI (kg/m^2^)	1.01	0.99–1.03	0.452	1.02	1.00–1.05	0.057
WHR	1.88	0.56–6.33	0.306	1.01	1.00–1.02	0.961

Abbreviations: BMI, body mass index; CI, confidence interval; OR, odds ratio; WHR, waist-to-hip ratio. * *p* < 0.05. ^a^ Adjusted for age, education, monthly income, marital status, self-reported health status, smoking status, and chewing betel nuts.

**Table 4:** Multivariate-adjusted ORs for the quartiles of happiness’ relations to health-related physical fitness measurements after adjustment for potential confounders.

Variables	Model 1 (Unadjusted)			Model 2 (Adjusted ^a^)		
	OR	95% CI	*p*	OR	95% CI	*p*
**Men (*n* = 27,930)**						
3 min step test						
>63.38	1.37	1.14–1.66	0.001 *	1.03	0.83–1.26	0.813
56.25–63.38	1.14	0.95–1.36	0.167	0.86	0.71–1.05	0.135
50.56–56.24	1.03	0.87–1.23	0.735	0.88	0.73–1.05	0.160
<50.56	1.00	—	—	1.00	—	—
1 min sit-up test (reps/min)						
>35.00	1.08	0.88–1.32	0.467	1.05	0.82–1.34	0.685
29.00–35.00	1.05	0.87–1.26	0.609	1.02	0.82–1.27	0.888
22.00–28.99	1.10	0.91–1.32	0.336	1.08	0.88–1.33	0.445
<22.00	1.00	—	—	1.00	—	—
Sit-and-reach test (cm)						
>29.00	1.36	1.13–1.64	0.002 *	1.11	0.91–1.36	0.312
21.00–29.00	1.40	1.17–1.67	<0.001 *	1.24	1.02–1.49	0.029 *
14.00–20.99	1.19	1.00–1.42	0.054	1.17	0.97–1.43	0.108
<14.00	1.00	—	—	1.00	—	—
BMI (kg/m^2^)						
<22.53	0.98	0.80–1.20	0.849	0.81	0.65–1.00	0.055
22.53–24.57	1.47	1.20–1.81	<0.001 *	1.01	0.81–1.26	0.925
24.58–26.81	1.08	0.90–1.30	0.394	0.78	0.64–0.95	0.014 *
>26.81	1.00	—	—	1.00	—	—
WHR						
<0.83	0.96	0.78–1.19	0.710	1.00	0.78–1.26	0.965
0.83–0.86	1.03	0.83–1.28	0.767	1.00	0.79–1.27	0.994
0.87–0.91	1.04	0.87–1.24	0.678	1.01	0.83–1.22	0.961
>0.91	1.00	—	—	1.00	—	—
**Women (*n* = 30,885)**						
3 min step test						
>62.07	1.26	1.04–1.54	0.021 *	0.90	0.72–1.12	0.332
54.88–62.07	1.22	1.00–1.48	0.049 *	0.92	0.75–1.14	0.450
49.45–54.87	1.12	0.92–1.36	0.270	0.97	0.78–1.20	0.755
<49.45	1.00	—	—	1.00	—	—
1 min sit-up test (reps/min)						
>25.00	1.43	1.14–1.79	0.002 *	1.48	1.12–1.95	0.006 *
19.00–25.00	1.24	1.02–1.52	0.036 *	1.32	1.04–1.67	0.025 *
10.00–18.99	1.19	0.98–1.45	0.079	1.28	1.03–1.60	0.028 *
<10.00	1.00	—	—	1.00	—	—
Sit-and-reach test (cm)						
>35.00	1.47	1.18–1.82	0.001 *	1.22	0.97–1.55	0.091
28.00–35.00	1.16	0.96–1.41	0.130	1.06	0.86–1.30	0.609
20.00–27.99	1.10	0.91–1.33	0.334	1.08	0.88–1.33	0.476
<20.00	1.00	—	—	1.00	—	—
BMI (kg/m^2^)						
<20.54	0.93	0.74–1.16	0.516	0.87	0.68–1.10	0.242
20.54–22.37	0.86	0.70–1.07	0.174	0.73	0.58–0.92	0.007 *
22.38–24.80	0.96	0.78–1.18	0.722	0.86	0.69–1.08	0.191
>24.80	1.00	—	—	1.00	—	—
WHR						
<0.76	0.84	0.67–1.05	0.131	0.95	0.74–1.22	0.684
0.76–0.79	0.90	0.71–1.13	0.366	0.96	0.75–1.24	0.771
0.80–0.85	0.86	0.70–1.04	0.126	0.89	0.72–1.10	0.289
>0.85	1.00	—	—	1.00	—	—

Abbreviations: BMI, body mass index; CI, confidence interval; OR, odds ratio; WHR, waist-to-hip ratio. * *p* < 0.05. ^a^ Adjusted for age, education, monthly income, marital status, self-reported health status, smoking status, and chewing betel nuts.
